# Опухолевый синдром в структуре нейрофиброматоза 1 типа у детей

**DOI:** 10.14341/probl13607

**Published:** 2025-12-02

**Authors:** В. С. Дерюгина, Ю. К. Тошина, И. Л. Никитина, А. М. Тодиева, Н. С. Дехтярева, Ю. В. Диникина

**Affiliations:** Национальный медицинский исследовательский центр им. В.А. АлмазоваРоссия; Almazov National Medical Research CenterRussian Federation

**Keywords:** дети, нейрофиброматоз 1 типа, феохромоцитома, плексиформная нейрофиброма, таргетная терапия, children, neurofibromatosis 1, pediatrics, pediatric oncology, plexiform neurofibroma, targeted therapy

## Abstract

Нейрофиброматоз 1 типа (НФ1) представляет собой мультисистемное генетическое заболевание, ассоциированное с повышенным риском развития опухолей в течение жизни. Наблюдение пациентов с НФ1 с привлечением специалистов различных профилей и применением программ скрининга является ключевым аспектом ранней диагностики характерных патологических состояний, определяющих риски инвалидизации и снижения продолжительности жизни. Авторами статьи представлен клинический случай пациентки подросткового возраста с НФ1 и сочетанными опухолевыми заболеваниями, при этом каждая из диагностированных неоплазий (плексиформная нейрофиброма и феохромоцитома) имела симптоматическое течение, приводя к серьезным нарушениям соматического статуса и качества жизни ребенка. Мультидисциплинарный подход в оказании медицинской помощи пациентке позволил достичь хороших результатов терапии с возобновлением привычного образа жизни, несмотря на продолжающуюся таргетную терапию.

## Abstract (en)

Neurofibromatosis type 1 (NF1) is a multisystem genetic disorder associated with a lifelong increased risk of tumor development. Comprehensive monitoring of patients with NF1, involving a multidisciplinary team of specialists and the implementation of screening programs, is crucial for the early detection of associated pathological conditions that contribute to disability and reduced life expectancy. This article presents a clinical case of an adolescent female patient with tumor manifestations of NF1, in whom both diagnosed neoplasms — plexiform neurofibroma and pheochromocytoma, had a symptomatic course, significantly affecting her physical health and quality of life. A multidisciplinary approach and correct treatment led to favorable outcomes, enabling the patient to resume a normal lifestyle, despite the continuation of targeted therapy.

## АКТУАЛЬНОСТЬ

Нейрофиброматоз 1 типа (НФ1) относится к синдромам предрасположенности к опухолевым заболеваниям с аутосомно-доминантным типом наследования [1]. В 50% случаев заболевание носит спорадический характер, что определяет возникновение патогенных вариантов гена NF1 de novo и вероятность рождения ребенка с НФ1 в семье здоровых родителей. Ген NF1 кодирует белок нейрофибромин, который экспрессируется во всех тканях организма и выполняет роль супрессора опухолевого роста, регулирующего функцию передачи сигналов биологического каскада RAS/Raf/MAPK [2]. При наличии патогенного варианта гена NF1 снижение количества и утрата функции нейрофибромина приводит к активации передачи сигнала по пути RAS/Raf/MAPK, тем самым увеличивая риски развития различных вариантов опухолей [3]. Частота встречаемости НФ1 в популяции соответствует 1 случаю на 2500–3000 человек и определяется как наиболее распространенный наследственный опухолевый синдром [1]. Диагноз «НФ1» устанавливается на основании сочетания клинических признаков согласно обновленным критериям диагностики от 2021 г. Молекулярно-генетическое тестирование является опциальным, используется в сомнительных клинических случаях или при наличии других показаний [6].

Опухолевый синдром при НФ1 как правило имеет возрастзависимый и прогрессирующий характер течения. Спектр характерных опухолей довольно широк, при этом для пациентов детского возраста свойственно преобладание доброкачественных новообразований (глиомы центральной нервной системы, плексиформные нейрофибромы, кожные/подкожные нейрофибромы) с увеличением вероятности развития злокачественных опухолей у взрослых [7][8]. Следует отметить, что именно опухолевые заболевания будут иметь наибольший вклад в снижение продолжительности жизни пациентов с НФ1. При этом невозможность прогнозирования их развития у каждого конкретного пациента подчеркивает значение программ мультидисциплинарного наблюдения и скрининга [7].

Феохромоцитома (ФХ) представляет собой катехоламинпродуцирующую опухоль, которая развивается из хромаффинных клеток мозгового вещества надпочечников и может иметь доброкачественный или злокачественный потенциал. В большинстве случаев ФХ характеризуется спорадическим характером развития, тем не менее до 25% случаев развиваются в структуре синдромов предрасположенности к опухолевым заболеваниям, включая НФ1, синдром Хиппеля-Линдау, множественной эндокринной неоплазии 2 типа. Риск развития ФХ у пациентов с НФ1 находится в пределах 0,1–5,7% [9][10]. Наиболее характерным возрастом возникновения ФХ является четвертое-пятое десятилетие жизни, тем не менее случаи таковой могут регистрироваться в другом, более раннем возрасте. Клиническая картина разнообразна и может варьировать от бессимптомного течения (случайная находка) до типичных патологических симптомов, нередко имеющих жизнеугрожающий характер [10][11]. Наиболее характерным является кризовое повышение систолического артериального давления (АД), сопровождающееся чувством страха, дрожью в теле, учащенным сердцебиением, профузным потоотделением [11]. Указанные симптомы могут быть неспецифичными и иметь многофакторный генез в структуре НФ1, что определяет необходимость своевременного адекватного обследования [7].

В данной статье представлен клинический случай сочетанных опухолевых заболеваний в структуре НФ1 у пациентки подросткового возраста. Нами представлен анамнез заболевания, особенности клинической картины, выбранная тактика и эффективность проведенного противоопухолевого лечения.

## ОПИСАНИЕ СЛУЧАЯ

Девочка М., 13 лет, с верифицированным диагнозом НФ1 была госпитализирована в отделение детской онкологии ФГБУ «НМИЦ им. В.А. Алмазова» с жалобами на рецидивирующие эпизоды повышения артериального давления, головокружение, тошноту и приступы удушья для обследования, определения тактики лечения.

Из анамнеза заболевания известно, что с первых дней жизни у пациентки были диагностированы пятна «по типу кофе с молоком» (рис. 1), в возрасте 1 месяца офтальмологом выявлен птоз справа, обусловленный образованием в толще правого верхнего века. В 3 года перенесла субтотальную радиоэксцизию новообразования, по результатам морфологического исследования установлен диагноз плексиформной нейрофибромы (ПН). В возрасте 8 лет по причине прогрессирующего птоза правого верхнего века перенесла повторную операцию по реконструкции экстраокулярных мышц без значимого эффекта. В 9 лет выполнена молекулярно-генетическая диагностика крови, по результатам которой обнаружен патогенный вариант гена NF1 — гетерозиготная (однонуклеотидная замена) мутация в 3 экзоне NF1. На основании клинических критериев диагностики и результатов генетического тестирования у пациентки верифицирован НФ1. В структуре НФ1 имел место идиопатический сколиоз грудопоясничного отдела позвоночника. В течение последующих двух лет активно продолжалось динамическое наблюдение у невролога, ортопеда, детского онколога по месту жительства. Принимая во внимание наличие неоперабельной симптоматической ПН правого верхнего века (рис. 2), были определены показания к назначению таргетной терапии МЕК 1/2 ингибитором селуметинибом. Терапия была инициирована в возрасте 11 лет.

**Figure:**
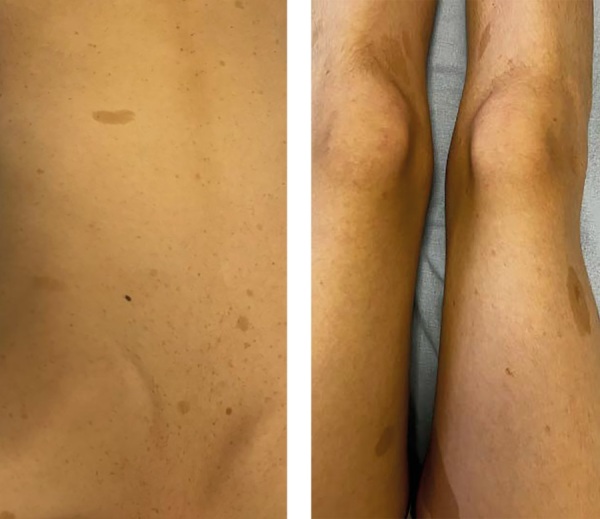
Рисунок 1. Множественные разнокалиберные пятна по типу «кофе с молоком» мультифокальной локализации.

**Figure:**
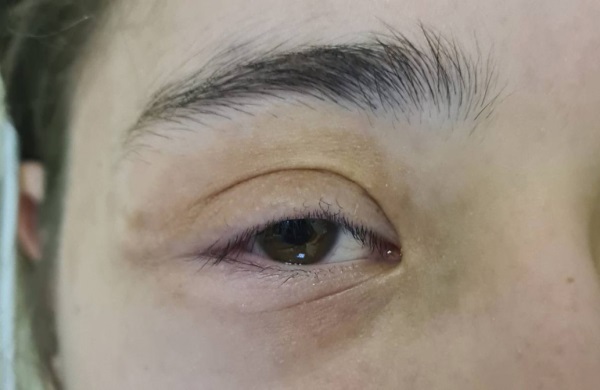
Рисунок 2. ПН в толще правого верхнего века, гиперпигментация кожи над образованием. Птоз.

Через 7 месяцев от начала таргетной терапии у девочки появились жалобы на преходящую головную боль, тошноту и рвоту. По результатам выполненной МРТ головного мозга причин, объясняющих вышеуказанные жалобы, не выявлено. Учитывая в анамнезе хронический поверхностный гастрит, дуоденит, дуоденогастральный рефлюкс, пациентка проходила обследование и лечение в отделении гастроэнтерологии по поводу обострения вышеперечисленных заболеваний, что не способствовало купированию вышеуказанных жалоб.

В связи с учащением приступов головной боли, сопровождавшихся рвотой, была экстренно госпитализирована в стационар по месту жительства. Была проведена МР-ангиография головного мозга, данных за патологию не получено. По результатам суточного мониторинга артериального давления диагностированы эпизоды выраженной артериальной гипертензии (АГ) с максимальными значениями АД до 170/122 мм рт.ст. в дневное время и 142/97 мм рт.ст. — в ночное. За время госпитализации проводилось симптоматическое лечение с применением ингибиторов АПФ (каптоприл 25 мг/сут) с умеренным эффектом. Девочка была выписана под наблюдение участкового педиатра.

При последующем наблюдении отмечались жалобы прежнего характера с отрицательной динамикой по степени тяжести АГ. В рамках очередного стационарного обследования по месту жительства по данным ультразвукового исследования (УЗИ) и МРТ брюшной полости в проекции левого надпочечника обнаружено гипоэхогенное новообразование размерами 9,3х7,3 см с наличием кистозного компонента размерами 2,6х1,9 см. Последующие этапы обследования и лечения выполнялись в условиях НМИЦ им. В.А. Алмазова.

За период госпитализации у пациентки неоднократно регистрировались эпизоды повышения АД, достигающие значений 190/100 мм рт.ст., сопровождавшиеся тошнотой, рвотой и головокружением. На фоне проводимой комбинированной антигипертензивной терапии (ингибиторы АПФ — капторил 25 мг/сут, блокаторы кальциевых каналов — 10 мг/сут) достигалось купирование эпизодов АГ. С целью определения дальнейшей тактики ведения были выполнены дополнительные визуализирующие исследования: мультиспиральная компьютерная томография органов брюшной полости с контрастным усилением (рис. 3), ПЭТ/КТ с ¹⁸F-ДОФА (рис. 4).

**Figure:**
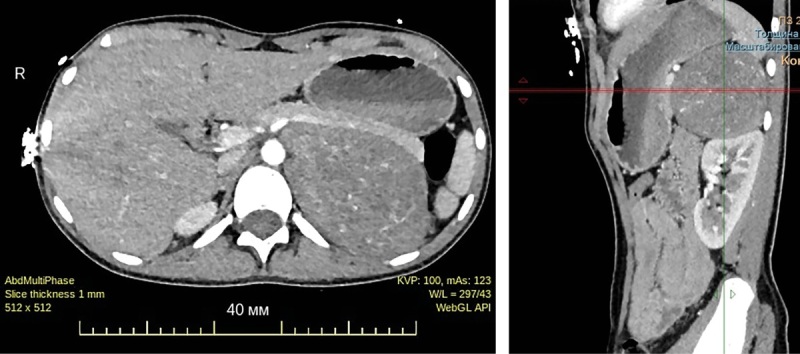
Рисунок 3. МСКТ органов брюшной полости. Определяется крупное образование левого надпочечника овоидной формы размерами 69х105х72 мм, неоднородной структуры за счет наличия единичных кист диаметром до 21 мм.

**Figure:**
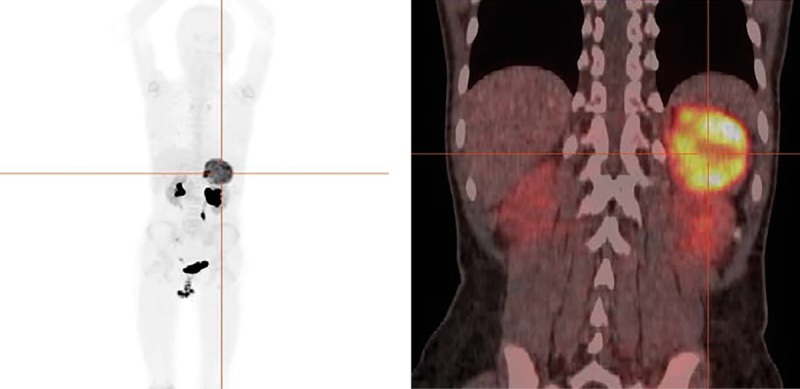
Рисунок 4. ПЭТ/КТ всего тела с 18F-ДОФА. В левом надпочечнике визуализируется ДОФА-позитивное образование размерами 105х67 мм и SUVmax=8,85, с выраженной неоднородной структурой за счет многочисленных разнокалиберных участков жидкостной плотности. Данных за наличие других патологических очагов нет.

По результатам исследования суточной мочи и плазмы крови на метанефрины и норметанефрины выявлено их значительное повышение, что указывало на наличие катехоламин-продуцирующей опухоли (табл. 1).

**Table:** Таблица 1. Показатели уровней метанефрина и норметанефрина в моче и плазме крови пациентки в динамике

Период исследования	Метанефрин	Норметанефрин
Моча	Плазма	Моча	Плазма
Возрастная норма	<0,053 мг/сут	<88 пг/мл	<0,068 мг/сут	<218,9 пг/мл
На момент госпитализации	2,083	7927,20	6,881	32191,20
Через 9 месяцев после операции	0,023	-	0,022	-

На основании полученных данных пациентке установлен предварительный диагноз: «ФХ с поражением левого надпочечника» и определены показания к оперативному лечению. С целью предоперационной подготовки, для предотвращения адренергических и гипертонических кризов, пациентка была переведена в отделение педиатрии, где в течение полутора недель проводилась лекарственная терапия с титрацией дозы препаратом из группы селективных конкурентных блокаторов постсинаптических альфа1-адренорецепторов доксазозином 0,25 мг 1 раз в сутки, с последовательной эскалацией до 3,5 мг 1 раз сутки. После достижения нормальных значений АД на фоне медикаментозной терапии пациентке была выполнена робот-ассистированная левосторонняя адреналэктомия. Осложнений интра- и послеоперационного периода отмечено не было, сохранялась нормотензия (90-110/54-70 мм рт.ст.), терапия доксазозином была отменена. По результатам морфологического исследования удаленного материала диагностирована умеренно дифференцированная ФХ. В послеоперационном периоде уровни метанефринов в суточной моче и плазме крови нормализовались (табл. 1), приступы АГ, тошноты, удушья, головокружения не рецидивировали.

В динамике, через 9 месяцев, госпитализирована в отделение педиатрии с целью оценки соматического статуса, контроля эффективности проведенной терапии. Состояние девочки расценено как удовлетворительное, активная, жалоб не предъявляла. За прошедший после оперативного удаления ФХ период пациентка прибавила в весе 10 кг. Также был констатирован старт менархе. Результаты лабораторного и инструментального обследования находились в пределах референсных значений.

По настоящее время пациентка продолжает получать терапию селуметинибом в отношении ПН верхнего века справа с коррекцией дозы с учетом переносимости. Продолжено регулярное динамическое наблюдение с привлечением мультидисциплинарной команды специалистов с целью мониторинга соматического здоровья, скрининга опухолевых и других, ассоциированных с НФ1, состояний.

## ОБСУЖДЕНИЕ

НФ1, как уже было отмечено, является мультисистемным заболеванием, с повышенными рисками развития в течение жизни опухолевых заболеваний широкого спектра [3][7]. При этом важно отметить, что именно таковые определяют высокую вероятность инвалидизации и снижения продолжительности жизни пациентов как за счет прогрессирующего опухолевого процесса, так и развития жизнеугрожающих осложнений. Одним из ключевых факторов прогноза будут являться своевременная диагностика онкологических заболеваний и адекватность оказываемой медицинской помощи.

В представленном нами клиническом случае у пациентки имело место развитие двух опухолей — ПН и ФХ, имеющих различные гистологическую принадлежность и возраст дебюта. Несмотря на доброкачественный характер обеих опухолей, каждая из них имела клинически значимое симптоматическое течение, приведшее к нарушению качества жизни ребенка и развитию спектра серьезных осложнений.

Следует отметить, что первые признаки ПН правого верхнего века появились еще в младенческом возрасте с осложнениями в виде правостороннего птоза, прогрессирующего косметического дефекта. Хирургические вмешательства проводились лишь в объеме частичного удаления ПН в силу ее локализации и диффузного роста, что не позволяло в полной мере купировать ассоциированные осложнения. Методы лекарственной терапии с доказанной эффективностью отсутствовали, в связи с чем выполнялось наблюдение за пациенткой до 11 лет, когда впервые была инициирована таргетная терапия селуметинибом. Эффективность проводимой системной терапии в течение последующих двух лет способствовала стабилизации роста ПН указанной локализации. В дальнейшем, несмотря на проводимый регулярный мониторинг пациентки, получающей таргетную терапию, при появлении патогномоничных для ФХ жалоб (повышение АД, сопровождавшееся головной болью, тошнотой, рвотой) адекватное обследование не выполнялось, что явилось причиной поздней верификации диагноза (через 11 месяцев от дебюта заболевания).

Не вызывает сомнений, что задержка диагностики является причиной прогрессирующего ухудшения состояния пациентов, развития тяжелых осложнений, а также рисков, ассоциированных с проведением хирургического вмешательства, как ключевого метода лечения ФХ [4]. Следует помнить и о вероятности бессимптомного течения ФХ, особенно на начальных этапах болезни, распространенность которых составляет около 10–17% [5]. При этом, по данным L. Képénékian и соавт., при НФ1 таковые составляют более 80%, а также имеют высокий риск злокачественного течения, что указывает на высокую роль программ скрининга у пациентов группы риска согласно международным рекомендациям [12]. Peduto и соавт. также отмечают важность определения генотип- фенотипических корреляций в понимании проявлений НФ1, что также может способствовать выявлению пациентов с высоким риском развития ФХ [2].

Ключевыми методами диагностики ФХ при наличии соответствующих клинических симптомов, являются лабораторные методы, включающие определение метанефринов и катехоламинов в моче и плазме крови, а также визуализирующие исследования, такие как КТ ОБП, МРТ ОБП, сцинтиграфия всего тела с ¹²³I-MIBG, ПЭТ-КТ с¹⁸F-FДОФА [7][13–15]. Следует отметить их высокую чувствительность и специфичность, позволяющую с высокой долей вероятности верифицировать диагноз и исключать метастатическое поражение [14][15]. В представленном нами случае было использовано несколько методов визуализации, включая анатомо-топографические (УЗИ, КТ, МРТ) и функциональные (ПЭТ-КТ с¹⁸F-FДОФА), что позволило в полном объеме оценить топографию новообразования, оценить степень метаболической активности (SUVmax=8,85) и исключить наличие отдаленных очагов метастазирования. Значимое повышение уровня метанефринов и норметанефринов в суточной моче и плазме крови, также подтверждало наличие катехоламин-продуцирующей опухоли, что соответствует результатам проводимых международных исследований о наличии корреляции результатов радионуклидной и лабораторной диагностики, отражающей биохимический фенотип ФХ [18].

Лечебные подходы при ФХ в первую очередь определяются стадией заболевания, при этом при локализованных формах используют хирургическое удаление новообразования [13][14]. Крайне важным аспектом является выполнение предоперационной подготовки, направленной прежде всего на адекватный контроль АД, предотвращение гипертонического криза интраоперационно, минимизацию рисков анестезии, поддержание стабильного АД во время операции и избежание гипотонии после удаления опухоли. Ключевая роль отводится использованию альфа-блокаторов, а после достижения должного эффекта — применению бета-блокаторов для контроля частоты сердечных сокращений [15][16][17]. Длительность предоперационной подготовки проводится в течение 7–30 дней, в среднем — 15 дней. Проводимая предоперационная подготовка пациентки в стационарных условиях с участием врачей педиатров и эндокринологов в полной мере способствовала медикаментозной нормализации АД, а также отсутствию интра- и послеоперационных осложнений.

Выбор хирургической техники при локализованных ФХ определяется анатомо-топографическими особенностями опухоли и ее размером [14]. Обоснованием для выбора робот-ассистированной левосторонней адреналэктомии являлось обеспечение минимально инвазивного подхода с меньшими рисками интра- и послеоперационных осложнений, более быстрым восстановлением пациентки. В ряде международных исследований отмечено, что использование робот-ассистированной адреналэктомии сокращает время операции, снижает риски повреждения капсулы опухоли, нерадикального удаления образования, объем кровопотери и частоту конверсии доступа к лапаротомии при опухолях надпочечников размером более 5 см, тем самым являясь предпочтительным малоинвазивным хирургическим методом удаления крупных опухолей надпочечников [19][20][21]. Следует отметить, что метастатические формы заболевания, помимо хирургического лечения, требуют проведения системной химиотерапии, в ряде случаев применения радиоизотопного лечения [13][14].

Окончательным методом верификации диагноза ФХ и определения дальнейшей тактики ведения является послеоперационное гистологическое исследование опухоли [14]. Так, в представленном случае был подтвержден диагноз умеренно дифференцированной ФХ левого надпочечника, что, принимая во внимание локальную стадию заболевания и радикальный объем проведенного оперативного вмешательства, не требовало использования системной противоопухолевой терапии. Пациентка оставлена под динамическим наблюдением многопрофильной команды специалистов с деликатным мониторингом соматического статуса, контролем переносимости проводимой таргетной терапии, назначенной по поводу ПН, а также выполнением скрининга ассоциированных с НФ1 заболеваний, что позволит своевременно реагировать на изменения в состоянии здоровья ребенка. За период наблюдения (14 месяцев) у пациентки сохраняется ремиссия ФХ, длительная стабилизация ПН на фоне приема селуметиниба, что позволяет девочке вести активный образ жизни.

## ЗАКЛЮЧЕНИЕ

На сегодняшний день лишь междисциплинарные подходы к ведению пациентов с мультисистемными заболеваниями, включая НФ1, могут обеспечить своевременность оказания медицинской помощи. В представленном клиническом случае у пациентки детского возраста с НФ1 и сочетанными опухолевыми проявлениями, а также ранним дебютом ФХ, нами продемонстрирована значимость онкологической настороженности специалистов всех профилей, необходимость соблюдения программ скрининга, а также ряд особенностей курации пациентов, определяющих исходы терапии.

## ДОПОЛНИТЕЛЬНАЯ ИНФОРМАЦИЯ

Источники финансирования. Работа выполнена по инициативе авторов без привлечения финансирования.

Конфликт интересов. Авторы декларируют отсутствие явных и потенциальных конфликтов интересов, связанных с содержанием настоящей статьи.

Участие авторов. Все авторы одобрили финальную версию статьи перед публикацией, выразили согласие нести ответственность за все аспекты работы, подразумевающую надлежащее изучение и решение вопросов, связанных с точностью или добросовестностью любой части работы.

Согласие пациента. Исследование выполнено в соответствии с Хельсинской декларацией ВМА в редакции 2013 г. Пациент добровольно подписал информированное согласие на публикацию персональной медицинской информации в обезличенной форме.
